# The effect of Self-Regulation Based Cognitive Psychoeducation Program on emotion regulation and self-efficacy in children diagnosed with attention deficit hyperactivity disorder

**DOI:** 10.1192/j.eurpsy.2024.951

**Published:** 2024-08-27

**Authors:** Z. Sökmen, S. Karaca

**Affiliations:** ^1^Department of Psychiatric Nursing; ^2^Marmara UniverDepartment of Psychiatric Nursing, Marmara University, Institute of Health Science, ISTANBUL, Türkiye

## Abstract

**Introduction:**

Attention deficit hyperactivity disorder (ADHD) is a neurodevelopmental disorder with early onset (Christiansen, H., et al. CPR 2019, 1–11), which is characterized by several symptoms, including lack of attention, hyperactivity, and impulsivity that are incompatible with age and developmental level (Caye, A., et al. 2020 JAACAP, 990–997)

**Objectives:**

This study aimed to determine the effect of Self-Regulation Based Cognitive Psychoeducation Program on emotion regulation and self-efficacy in children diagnosed with attention deficit hyperactivity disorder (ADHD) and receiving medication.

**Methods:**

The sample of this study with control group and pre-test, post-test and follow-up randomized experimental design consisted of children followed in the child and adolescent mental health outpatient clinic of a state hospital. The data were evaluated by parametric and non-parametric analyses.

**Results:**

A statistically significant increase was determined in the internal functional emotion regulation mean scores of children, who participated in the Self-Regulation Based Cognitive Psychoeducation Program, measured before, immediately after, and 6 months after the intervention (p < 0.05). A statistically significant increase was also found in their external functional emotion regulation mean scores measured before and 6 months after the intervention (p < 0.05). In addition, a statistically significant difference was found between their internal dysfunctional and external dysfunctional emotion regulation mean scores measured before and 6 months after the intervention; however the mean scores of those in the control group 6 months after the intervention were higher than those in the intervention group (p < 0.05). Furthermore, there was a statistically significant increase in their self-efficacy mean scores measured before and 6 months after the intervention (p < 0.05).

**Conclusions:**

The Self-Regulation Based Cognitive Psychoeducation Program was found be effective in increasing the levels of emotion regulation and self-efficacy in children with ADHD.

**Disclosure of Interest:**

None Declared

